# The Complex Interactions Between Rotavirus and the Gut Microbiota

**DOI:** 10.3389/fcimb.2020.586751

**Published:** 2021-01-08

**Authors:** Andrew HyoungJin Kim, Michael P. Hogarty, Vanessa C. Harris, Megan T. Baldridge

**Affiliations:** ^1^ Department of Medicine, Division of Infectious Diseases, Washington University School of Medicine, St. Louis, MO, United States; ^2^ Department of Medicine, Division of Infectious Diseases and Department of Global Health (AIGHD), Amsterdam University Medical Center, Academic Medical Center, Amsterdam, Netherlands

**Keywords:** rotavirus (human and animal), microbiota, immunity, rotavirus vaccine, animal models, *in vitro* models

## Abstract

Human rotavirus (HRV) is the leading worldwide cause of acute diarrhea-related death in children under the age of five. RV infects the small intestine, an important site of colonization by the microbiota, and studies over the past decade have begun to reveal a complex set of interactions between RV and the gut microbiota. RV infection can temporarily alter the composition of the gut microbiota and probiotic administration alleviates some symptoms of infection *in vivo*, suggesting reciprocal effects between the virus and the gut microbiota. While development of effective RV vaccines has offered significant protection against RV-associated mortality, vaccine effectiveness in low-income countries has been limited, potentially due to regional differences in the gut microbiota. In this mini review, we briefly detail research findings to date related to HRV vaccine cohorts, studies of natural infection, explorations of RV-microbiota interactions in gnotobiotic pig models, and highlight various *in vivo* and *in vitro* models that could be used in future studies to better define how the microbiota may regulate RV infection and host antiviral immune responses.

## Introduction

Prior to the introduction of rotavirus vaccines (RVVs) in 2006, human rotavirus (HRV) was the leading global cause of mortality due to acute gastroenteritis in children under the age of five (Rodriguez et al., 1980; Tate et al., 2016). Despite successful implementation of the vaccine, RV still accounts for the higest numbers of death due to gastroenteritis globally. RV is a double-stranded RNA virus in the *Reoviridae* family. RV virions are non-enveloped and composed of three concentric protein layers, which contain a genome of 11 segments of dsRNA encoding 6 structural proteins and 6 non-structural proteins (Desselberger, 2014). RV predominantly targets mature enterocytes in the small intestine, and infection is regulated by both innate and adaptive immune responses (Greenberg and Estes, 2009). The RVV has significantly decreased HRV-associated mortality rates but both the efficacy and effectiveness of RVV in preventing severe gastroenteritis in children is significantly lower in low-income vs high-income settings (Parashar et al., 2003; O’Ryan, 2017). As one of the possible etiologies contributing to this variation in RVV effectiveness, a number of recent studies have suggested a potential role for the commensal gut microbiota in regulating RVV responses (Harris et al., 2017a; Harris et al., 2017b; Harris, 2018; Harris et al., 2018). This review details what is currently understood about the complex interactions between RV infection and immunity and the gut microbiota, summarizing the evidence to date as well as clarifying the *in vitro* and *in vivo* systems available to explore these interactions.

## Interactions Between Human Rotavirus and the Gut Microbiota

HRV infects the intestine, which also hosts the human body’s largest collection of microorganisms. As understanding of the broad physiological importance of the commensal gut microbiota has grown (Kennedy et al., 2018), so has interest in its interactions with pathogens. Enteric viruses, including RV, norovirus, adenovirus and astrovirus, induce diarrhea, which can alter the human gut microbiome by shifting the dominant phylum from Bacteroidetes to Firmicutes, decreasing bacterial diversity and increasing opportunistic pathogens, such as the genera *Shigella* (Ma et al., 2011). HRV-induced gastroenteritis has been specifically shown to temporarily decrease the diversity of and significantly alter the microbiota composition after infection, though recovery is associated with return to a level of diversity that reflects the non-infected state (Chen et al., 2017; Dinleyici et al., 2018). In addition to broad phylogenetic changes, species-specific shifts after HRV infection have also been reported, such as a transition from *Bacteroides vulgatus* and *stercoris* to *Bacteroides fragilis*, suggesting structural changes to the gut microbiota at all taxonomic levels (Zhang et al., 2009). Thus, HRV infection and associated diarrheal illness clearly affect the intestinal bacterial microbiota. Importantly, however, there is increasing appreciation that the interactions between HRV and the gut microbiota are bidirectional, and that the gut microbiota can also influence the intensity and duration of HRV infection (Saavedra et al., 1994; Fang et al., 2009; Teran et al., 2009; Grandy et al., 2010; Huang et al., 2014; Lee et al., 2015).

There have been persistent efforts to define the effects of the gut microbiota on human RV infection. Several clinical trials, either conducted pre-RVV in the United States or post-RVV in Bolivia, Taiwan, and Korea, have investigated the effects of probiotic administration on infants and children with natural symptomatic RV infection (Saavedra et al., 1994; Fang et al., 2009; Teran et al., 2009; Grandy et al., 2010; Huang et al., 2014; Lee et al., 2015). In these studies, probiotics including *Lactobacillus rhamnosus*, *Saccharomyces boulardii*, and *Bifidobacterium longum* resulted in mild to moderate reduction of RV-associated symptoms, such as duration of diarrhea and fecal RV levels. However, another prospective, randomized, double-blind trial conducted post-RVV in the United States failed to identify a protective effect for *L. rhamnosus* GG in protecting children against acute gastroenteritis (Schnadower et al., 2018). Probiotic administration, in conjunction with traditional treatment such as oral rehydration, may thus have potential as a therapeutic intervention for RV-induced gastroenteritis in some settings, but the mechanisms by which these bacterial taxa affect RV infection in humans remain unclear. Studies using an *in vivo* model of HRV infection have the potential to shed additional light on these interactions.

Neonatal gnotobiotic pigs recapitulate many physiological factors of human infants (Meurens et al., 2012) and are susceptible to HRV infection (Saif et al., 1996), making them a useful animal model to investigate the interactions between the gut microbiota and HRV (Yuan and Saif, 2002; Zhang et al., 2008a; Kandasamy et al., 2016; Paim et al., 2016; Kumar et al., 2018). Transplantation of the gut microbiota of human infants to neonatal gnotobiotic pigs permitted evaluation of the impact of diet on both the gut microbiota composition and HRV disease severity (Kumar et al., 2018). Post-transplant, the piglets shared the majority of bacterial taxa identified in the original sample, and colonized piglets were observed to have reduced HRV-induced diarrhea and viral shedding compared to their noncolonized germ-free counterparts. In addition, a protein-sufficient diet further limited the severity of infection compared to a protein-deficient diet, suggesting that proper nutrition can also be protective, potentially *via* maintenance of the microbiota (Kumar et al., 2018). Similar to the human cohort studies discussed above, neonatal gnotobiotic pigs have been used to test the effects of various probiotics such as *Escherichia coli* Nissle 1917, *Lactobacillus rhamnosus* GG, *Lactobacillus acidophilus*, and *Lactobacillus reuteri* on HRV infection (Zhang et al., 2008a; Kandasamy et al., 2016; Paim et al., 2016). In general, these studies have supported the beneficial effects of probiotic use in limiting the symptoms of HRV infection or enhancing B cell responses. *E. coli* Nissle administration has been shown to reduce diarrhea severity and HRV shedding in pigs by both increasing IL-6, IL-10, and IgA levels as well as potentially directly binding RV particles (Kandasamy et al., 2016). Furthermore, *E. coli* Nissle altered gene expression in several enteric cell types and reduced enterocyte proliferation, implicating the probiotic in reducing barrier disruption and maintaining the absorptive function of the gut (Paim et al., 2016). In contrast, colonization of germ-free neonatal pigs with lactic acid bacteria probiotics alone was insufficient to promote intestinal B cell responses (Zhang et al., 2008a). An important caveat of these studies is that though neonatal pigs recapitulate many aspects of HRV infection in human infants, there is always the possibility that probiotic effects observed are unique to piglets. Since enhancement of immune responses is key for these potential therapeutic interventions, further investigations are needed to fully resolve the effect of these probiotics on HRV immune responses.

## Interactions Between Rotavirus Vaccines and the Gut Microbiota

Rotarix and Rotateq, two different types of live-attenuated oral RVVs, are currently the most studied RVV. Rotarix is a monovalent, attenuated vaccine derived from HRV strain G1P[8] (Ward and Bernstein, 2009) and licensed by the FDA with a dosage regimen of two oral doses at 6 and 10 weeks of age (Payne et al., 2011). Rotateq is a pentavalent human-bovine vaccine containing five RV reassortants derived from human and bovine viral species (Vesikari et al., 2009) and licensed by the FDA with a dosage regimen of three oral doses at 2, 4, and 6 months of age (Payne et al., 2011). In high-income settings, both vaccines show over 85% efficacy in preventing severe RV gastroenteritis in infants, and effectively increase anti-RV serum IgA, which is highly correlated with RVV efficacy (Vesikari et al., 2006; Patel et al., 2013). Prior studies, examining phylum-level differences, indicate RVV administration does not broadly affect the microbiota (Garcia-Lopez et al., 2012; Ang et al., 2014).

**Figure 1:**
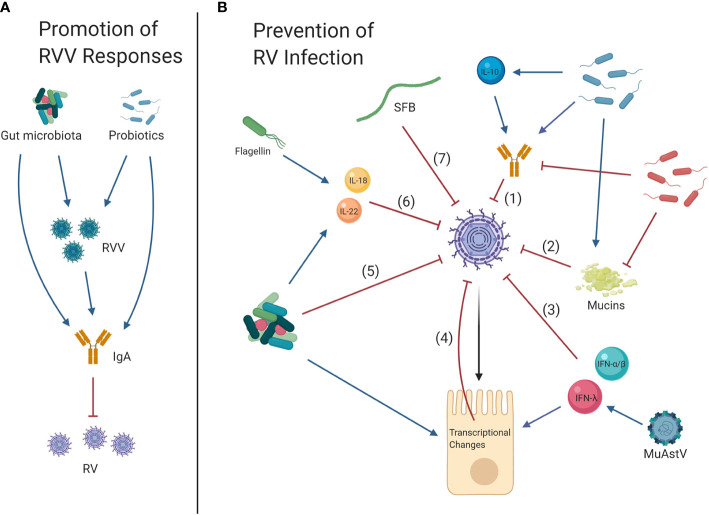
Effects of the gut microbiota on rotavirus vaccines (RVV) and rotavirus (RV) infection. **(A)** Promotion of RVV Responses: Gnotobiotic pig studies have suggested that overall, the gut microbiota and specific probiotics promote RVV efficacy and the development of protective IgA responses, which in turn limit future RV infection. **(B)** Prevention of RV infection: Clinical trials as well as studies in gnotobiotic pigs and neonatal rats have shown that the commensal microbiota and probiotics may also reduce symptoms of RV infection, partially through development of increased IL-10 levels and anti-RV IgA (1). Murine and gnotobiotic neonatal rat models have shown probiotics may reduce RV infection by inducing mucin secretion (2). Alternately, some studies in mice have shown that bacteria may facilitate RV infection by limiting anti-RV IgA responses and degrading mucins which potentially prevent RV-cell attachment (1, 2). Cytokines type I (IFN-α/β) and III IFNs (IFN-λ) are also antiviral (3), and virome element murine astrovirus (MuAstV) can protect against RV by inducing IFN-λ, thereby upregulating interferon stimulated genes (ISG) (4). Gnotobiotic pig studies have indicated that probiotics can inhibit RV infection and reduce disturbance of the intestinal barrier as observed through increased *Villin*, *Muc2*, *CgA*, and *Pcna* and decreased *Sox9* expression, indicating restoration of differentiated enterocyte, goblet, enteroendocrine and transient amplifying progenitor cell function and decreased proliferation of stem cells, respectively (4). Bacteria can also directly interact with RV particles, which may reduce infection (5). Studies in mice have shown that elements of the gut microbiota including bacterial flagellin can activate IL-18 and IL-22 signaling to protect against RV (6). Segmented filamentous bacteria (SFB) can also prevent RV infection through immune cell-independent mechanisms (7).

In contrast, a number of recent studies exploring species-level differences have implicated the gut microbiota in regulating RVV efficacy. The gut microbiota factors responsible for the variation in RVV efficacy have been explored in various clinical studies with cohorts from different regions including Ghanaian, Pakistani, Finnish, Indian, and Nicaraguan infants (Isolauri et al., 1995; Harris et al., 2017a; Harris et al., 2017b; Harris et al., 2018; Lazarus et al., 2018; Parker et al., 2018; Fix et al., 2020). These studies suggest that geographical differences in gut bacterial composition may contribute to RVV efficacy, and indeed specific bacterial taxa have been associated with RVV responses, including a positive correlation for *Streptococcus bovis* and a negative correlation with members of the Bacteroidetes phylum with anti-RV IgA responses (Harris et al., 2017a; Harris et al., 2017b) (**Figure 1A**). In other cohorts, no association of specific microbiota taxa with seroconversion was found. These discrepancies may derive from differences in microbiome sequencing methodologies, or in distinct gut microbiota composition influenced by different geographical regions (Parker et al., 2018; Fix et al., 2020). A proof-of-principle study tested whether prospective alteration of the microbiota with narrow-spectrum (vancomycin alone) or broad-spectrum (vancomycin, ciprofloxacin and metronidazole) antibiotics could modulate RVV immunogenicity in adults. Significantly decreased levels of Firmicutes and increased level of Proteobacteria in the narrow-spectrum group correlated with enhanced anti-RV IgA, but not IgG, titer boosting as well as increased RVV shedding (Harris et al., 2018). However, the specific mechanism of how bacterial taxa may regulate RV immune responses, including anti-RV IgA, has not yet been defined.

Currently available oral rotavirus vaccine efficacy is generally considered comparable across low- and middle-income settings. Increasing numbers of generic oral vaccines are available on the market and they differ in terms of number of doses, timing of doses, and cost. Choice of vaccines in low- and middle-income settings is often driven by cost, support from the Global Alliance for Vaccines and Immunization, feasibility of integration with existing vaccination programs, and availability. There are many regional factors including maternal antibodies, viral co-infection, host genetics, diet, and other geographic differences that may contribute to diminished RVV protection in low- and middle-income settings along-side the microbiota. Currently, all human studies evaluating correlations between RVV and intestinal microbiota have evaluated RVV immunogenicity and not vaccine efficacy against severe RV gastroenteritis. RV immunogenicity, as measured by anti-RV IgA is an imperfect correlate of protection and may not reflect protection from clinically relevant disease. Additional region-specific investigations are therefore needed, evaluating the microbiota’s correlation with vaccine efficacy against severe RV gastroenteritis in order to clarify the effects of the gut microbiota on RVV performance, with subsequent validation in *in vivo* models (Burke et al., 2019).

Gnotobiotic pigs have also been used to examine the effect of the gut microbiota on RVV efficacy and vice-versa (Zhang et al., 2008b; Vlasova et al., 2013; Kandasamy et al., 2014; Zhang H. et al., 2014; Twitchell et al., 2016). Enhanced cell-mediated immunity, as measured by more RV-specific IFN-γ producing T cells, in response to RVV was observed in neonatal gnotobiotic pigs transplanted with healthy infant gut microbiota compared to an unhealthy infant gut microbiota, though RV-specific IgA, IgG, and virus neutralizing antibody responses were unaffected. (Twitchell et al., 2016). *L. rhamnosus* GG administration to pigs, which had also received transplanted human gut microbiota and RVV, prevented the phylum-level shift from Firmicutes to Proteobacteria caused by HRV challenge, but had no significant effect on HRV infection responses (Zhang H. et al., 2014). In contrast, other reports suggest that colonization of neonatal gnotobiotic pigs with *L. rhamnosus* GG and *Bifidobacterium animalis lactis* Bb12, followed by RVV vaccination, significantly enhances RVV immunogenicity and diminishes HRV infection responses including severe diarrhea and viral shedding (Vlasova et al., 2013; Kandasamy et al., 2014) (**Table 1**). However, these results were not fully replicated in human clinical trials, wherein *L. rhamnosus* GG treatment had a positive but very modest or no significant effect on RVV immunogenicity (Isolauri et al., 1995; Lazarus et al., 2018). The factors responsible for diminished probiotic effectiveness in humans remain unresolved, and additional studies involving transplantation of human infant gut microbiota in neonatal gnotobiotic pigs may help to resolve which factors will help drive immunologically relevant responses in humans.

**Table 1:** Effects of microbiota and probiotics, observed in individual studies, on HRV/RVV immune responses in gnotobiotic neonatal pigs.

Study details	Experimental design	Gut microbiota or probiotic source	Diarrheal severity	HRV fecal shedding	Innate Immune Reponses	Humoral Immunity	T cell Responses
Zhang et al., 2008b	Vac+Pro (vaccinated, probiotics) Vac (vaccinated, no probiotics) Pro (unvaccinated, probiotics) Control (unvaccinated, no probiotics)	*Lactobacillus acidophilus* strain NCFM	▼in Vac+Pro compared to Vac	no difference		▲IgA and IgG antibody-secreting cell responses in ileum, and serum IgM, IgA and IgG antibody and virus neutralizing antibody titers in Vac+Pro compared to Vac	▲ HRV-specific IFN-γ producing CD8+ T cell responses in ileum and spleen in Vac+Pro compared to Vac
Vlasova et al., 2013	Vac+Pro (vaccinated, probiotics) −/+ HRV challenge Vac (vaccinated, no probiotics) −/+ HRV challenge Pro (unvaccinated, probiotics) −/+ HRV challenge Control (unvaccinated, no probiotics) −/+ HRV challenge	*Lactobacillus rhamnosus* strain GG *Bifidobacterium animalis lactis* strain Bb 12	▼in all groups + HRV compared to Control + HRV	▼in all groups + HRV compared to Control + HRV	▲ pDC/cDC and MHCII+ pDC/cDC in ileum and blood of Pro + HRV & Vac+Pro + HRV compared to Vac + HRV and Control +HRV ▲ serum IFN-alpha levels in Controls + HRV compared to all other groups + HRV ▼ TLR 2 and TLR4 expressing MNCs from Vac+Pro - HRV ▲ TLR3 expressing MNCs from Pro + HRV. ▲ CD4, SWC3a, CD11R1, MHCII expressing intestinal and blood MNCs in Pro + HRV & Vac+Pro + HRV compared to Vac + HRV and Control +HRV		
Kandasamy et al., 2014	Vac+Pro (vaccinated, probiotics) −/+ HRV challenge Vac (vaccinated, no probiotics) −/+ HRV challenge Pro (unvaccinated, probiotics) −/+ HRV challenge Control (unvaccinated, no probiotics) −/+ HRV challenge	*Lactobacillus rhamnosus* strain GG *Bifidobacterium animalis lactis* strain Bb 12	▼in Vac+Pro + HRV compared to Vac + HRV	▼in Vac+Pro + HRV compared to Vac + HRV	▲ mature conventional DCs and plasmacytoid DCs in Vac+Pro + HRV compared to Vac + HRV ▲ileal MNCs IL-6 in Pro −/+ HRV & Vac+Pro −/+ HRV compared to Vac −/+ HRV & Control −/+ HRV ▲ileal MNCs IL-10 in Vac+Pro -HRV compared to Vac - HRV	▲ small intestinal anti-HRV IgA and total IgA titers in Vac+Pro + HRV compared to Vac + HRV ▲ duodenal HRV IgA antibody-secreting cell response in Vac+Pro + HRV compared to Vac +HRV ▼ serum anti-HRV IgG antibody and total IgG titers in Vac+Pro −/+ HRV compared to Vac −/+ HRV ▲ CD21+CD2- B cells in ileum and duodenum in Vac+Pro + HRV compared to Vac + HRV	
Zhang H. et al., 2014	Healthy stool, vaccinated −/+ HRV challenge Healthy stool +Pro, vaccinated −/+ HRV challenge	*Lactobacillus rhamnosus strain GG* Healthy infant stool	no difference	no difference			
Twitchell et al., 2016	Healthy stool, vaccinated −/+ HRV challenge Unhealthy stool, vaccinated −/+ HRV challenge	Healthy and unhealthy (high enteropathy score with minimal seroconversion to RVV) Nicaraguan infant stool	▼ in Healthy stool + HRV vs Unhealthy stool + HRV	▼ in Healthy stool + HRV vs Unhealthy stool + HRV		no difference in anti-HRV IgA, IgG and virus neutralizing antibody between groups	▲ileum, spleen and blood virus-specific T cell immune response in Healthy - HRV compared to Unhealthy - HRV

### Models for Studying Rotavirus Infection and Immunity

Beyond gnotobiotic pig models described above, murine models have been beneficial to understand the mechanisms through which the microbiota interacts with RV (**Figure 1B**). Multiple strains of murine rotavirus (mRV) are available, and adult mice are susceptible to infection. However, only mice under 14 days of age develop diarrhea (Greenberg and Estes, 2009), which is a significant means by which infection can alter the intestinal microbiota (Ma et al., 2011). BALB/c mice have been shown to be approximately 1,000 times more susceptible to infection than C57BL/6 mice, suggesting important, and still unclear, mechanisms of genetic regulation of susceptibility (Blutt et al., 2012).

MRV infection causes significant changes to the composition of the ileal microbiota by inducing mucin secretion from goblet cells (Engevik et al., 2020). This compositional shift favors mucin-degrading bacteria *Bacteroides* and *Akkermansia*, which in turn could also promote mRV infection *in vitro*. Furthermore, ampicillin and neomycin administration has been linked to protection against mRV infection and symptoms *via* generation of a more robust humoral/mucosal response, further implicating the microbiota in promoting mRV infection (Uchiyama et al., 2014).

Conversely, probiotics *Bifidobacterium bifidum*, *Bifidobacterium dentium*, and *Bifidobacterium longum* mediate protective effects against mRV infection, potentially *via* increased mucin secretion, which can prevent efficient RV cell attachment (Chen et al., 1993; Duffy et al., 1994a; Duffy et al., 1994b; Boshuizen et al., 2005; Munoz et al., 2011; Kawahara et al., 2017; Engevik et al., 2019). Microbes such as *Lactobacillus reuteri* and *Bifidobacterium* species can also hamper infection by increasing mRV-specific IgA levels (Qiao et al., 2002; Preidis et al., 2012). Recently, segmented filamentous bacteria (SFB) have been found to inhibit mRV infection in an immune cell-independent manner, possibly by changes in host gene expression, accelerated epithelial cell turnover, and/or direct neutralization (Shi et al., 2019).

Regulation of critical antiviral cytokine pathways is an important mechanism for microbiota-mediated regulation of RV. Bacterial flagellin mediates antiviral effects *via* toll-like receptor 5 and NOD-like receptor C4-mediated activation of cytokines interleukin-22 (IL-22) and IL-18, which protect against RV infection (Zhang B. et al., 2014). IL-22 has been shown to be profoundly antiviral against RV, especially in combination with mucosal antiviral cytokine interferon-lambda (IFN-λ) (Hernandez et al., 2015). Type I (IFN-α/β) and III IFNs (IFN-λ) are induced by RV and exert age-dependent antiviral effects against RV (Broquet et al., 2011; Pott et al., 2011; Lin et al., 2016; Ingle et al., 2018). We recently found that a non-bacterial element of the microbiota, specifically chronic murine astrovirus infection in immunocompromised mice, stimulates high levels of IFN-λ, but not type I or II IFNs, to protect mice against RV infection (Ingle et al., 2019), indicating that the virome can have important interactions with RV as well. Murine models of RV are thus helpful for clarifying mechanisms and molecular pathways by which the microbiota can promote or prevent infection.

Neonatal rats have also been used as an animal model to study effects of probiotics on RV infection. Rats can be readily infected using simian RV (SRV) strain SA-11, rare reassortants of which have been shown to infect humans (Ciarlet et al., 2002; Awachat and Kelkar, 2005; Perez-Cano et al., 2007), and both viremia and extraintestinal spread has been observed in rats infected with HRV or SRV (Crawford et al., 2006). In neonatal rats, probiotic LGG administration reduces SRV viral levels in serum and colon samples (Ventola et al., 2012). Furthermore, both live and dead LGG administration ameliorate the poor weight gain and colon swelling associated with SRV (Ventola et al., 2012). Gnotobiotic neonatal rats fed fermented milk containing probiotic *Lactobacillus casei* DN-114 001 exhibit reduced clinical measures of diarrhea, decreased vacuolation in intestinal epithelial cells, and decreased numbers of sulfated mucin containing cells (Guerin-Danan et al., 2001). If the rate of mucin secretion exceeds the rate of production then the absence of mucin containing cells are indicative of increased mucin secretion, a mechanism through which probiotics inhibit RV infection in mice (Kawahara et al., 2017). *Bifidobacterium breve* M-16V has also been shown to reduce SRV-induced diarrheal severity and duration (Rigo-Adrover et al., 2017; Rigo-Adrover et al., 2018). In the absence of RV infection, *B. breve* M-16V is sufficient to enhance IgA production, which may play a role in symptom reduction (Rigo-Adrover et al., 2016). A prebiotic mixture of short chain galactooligosaccharides and long chain fructooligosaccharides alone or in conjunction with *B. breve* M-16V also reduces SRV diarrheal severity, duration and viral shedding, enhances early serum anti-RV IgG and intestinal anti-RV IgA responses, and increases IL-4 and IL-10, both of which reduce RV infection in animal models (Gandhi et al., 2017; Rigo-Adrover et al., 2017; Rigo-Adrover et al., 2018). Thus, data from neonatal rat models supports protective effects of probiotics against RV.

Despite multiple human clinical studies suggesting interactions between the gut microbiota and HRV infection, the cellular implications of these interactions remain unclear, supporting the utility of *in vitro* models. Bacterially produced or modified metabolites may be critical for mediating the effects of the microbiota on RV, and can be readily tested using cell culture systems such as Caco2 or MA104 cells. Inhibitory effects of bile acids, which are regulated by the microbiota, on RV replication through activation of the farnesoid X receptor have been observed in both cell lines and mice (Kim and Chang, 2011). Indeed, exploration of how the metabolome, or the combined set of metabolites, present in the gut regulates RV infection will be an important area of future study.

Human intestinal organoids (HIOs) and enteroids (HIEs), which respectively use human induced pluripotent stem cells or culturing of epithelial crypt domains *ex vivo*, represent potentially useful systems for these studies (Kim and Chang, 2011; Yin et al., 2015; Saxena et al., 2016; Yin et al., 2016; Saxena et al., 2017). HIOs have been successfully used to cultivate HRV for analysis of antivirals (Finkbeiner et al., 2012; Yin et al., 2015; Yin et al., 2016), and HRV cultivation in HIEs has been used to study cellular mechanisms of HRV-induced diarrhea (Saxena et al., 2016) and to explore innate immune responses to HRV (Saxena et al., 2017). These systems thus hold promise for exploration of HRV-microbiota interactions (Blutt et al., 2018), though challenges in co-culturing eukaryotic cells and anaerobic gut microbes are non-trivial. Technical advances in replicating intestinal conditions *in vitro* may make these explorations increasingly feasible (Karve et al., 2017; Jalili-Firoozinezhad et al., 2019; Shin et al., 2019), hopefully yielding future insight into the mechanisms of how the gut microbiota affects HRV.

## Future Directions

Studies thus far support critical interactions between the host gut microbiota and RV infection as well as development of effective immune responses to RVV. However, much is still unknown about the nature of these interactions. Key remaining questions include: What are the key endogenous bacterial taxa influencing HRV infection and RVV responses? Do these taxa mediate effects *via* direct interactions with RV, modulation of the host epithelium, or regulation of host cytokine pathways? Can pre-, pro-, or postbiotic (nutritional, bacterial, or bacterial product/metabolite) interventions be improved to limit severity of HRV infection or enhance RVV responses?

Continued and expanded use of mouse, pig, and HIO/HIE models will be critical to further elucidate mechanisms of RV and microbiota interactions. Specific bacterial taxa, or other microbiota elements, that modulate infection and immune responses would be useful to identify in all models, as species-specific taxa may still mediate parallel effects on RV to provide common mechanistic insights. Further careful exploration of geographically diverse human cohorts in the context of natural HRV infection and RVV administration will also be critical to understand the complex environmental factors, including the microbiota, at play. Finally, continuous discourse between the human clinical study arena and experimental models to carefully test hypotheses will be key to advancing our capacity to combat RV infection in the future.

## Author Contributions

AK, MH, and MB wrote and edited the manuscript. VH edited the manuscript. All authors contributed to the article and approved the submitted version.

## Funding

MB was supported by NIH grants R01 AI141716 and R01 OD024917, a Children’s Discovery Institute of Washington University and St. Louis Children’s Hospital Interdisciplinary Research Initiative grant (MI-II-2019-790), and The Mathers Foundation. AK was supported by T32 AI007163. MH was supported by a stipend from Washington University School of Medicine through the BioSURF program.

## Conflict of Interest

The authors declare that the research was conducted in the absence of any commercial or financial relationships that could be construed as a potential conflict of interest.
